# Ectopic Endometriosis Seeded to the Rectus Muscle

**DOI:** 10.7759/cureus.5873

**Published:** 2019-10-09

**Authors:** Gabriel O Ologun, Cara Hannigan, Edmund Kwarteng, Aaron Bambil, David Bertsch

**Affiliations:** 1 General Surgery, Robert Packer Hospital/Guthrie Clinic, Sayre, USA; 2 Family Medicine, Caribbean Medical University, Cupecoy, CUW; 3 Surgical Oncology, Robert Packer Hospital/Guthrie Clinic, Sayre, USA

**Keywords:** endometriosis, scar endometriosis, hormonal suppression, rectus muscle, abdominal mass

## Abstract

Endometriosis is a pathological condition whereby endometrial glands and stroma are located outside the physiologically expected location, the uterus. A lesion is usually located in the pelvis but can also occur in the diaphragm, bowel, pleural cavity, or surgical scar. It is a benign estrogen-dependent condition. Here, we present the case of a 40-year-old woman with ectopic endometriosis.

## Introduction

Surgical scar endometriosis is a rare condition that is very difficult to diagnose because of its nonspecific symptoms. The patient may present with pain at the incision site from previous surgery (abdominal pain in the region of the scar). The pain may be cyclical, intensifying during the menstrual period. Some patients may present, in addition to the symptoms above, with some leakage from the scar area [1]. Here, we present the case of a 40-year-old woman with ectopic endometriosis. Informed consent was obtained.

## Case presentation

A 40-year-old woman, who has had one pregnancy and has delivered once (G1P1), was referred by her gynecologist to our surgical oncology clinic for evaluation of a lower midline abdominal wall mass. The patient reported having abdominal pain and discomfort in this area for more than five years, with no definite sense of lump or mass, and worsening pain immediately prior to and during her menses. Personal history revealed that she had a planned lower segment cesarean section several years ago. She was recently diagnosed with dysfunctional uterine bleeding. She is not taking any medication at home.

**Figure 1:**
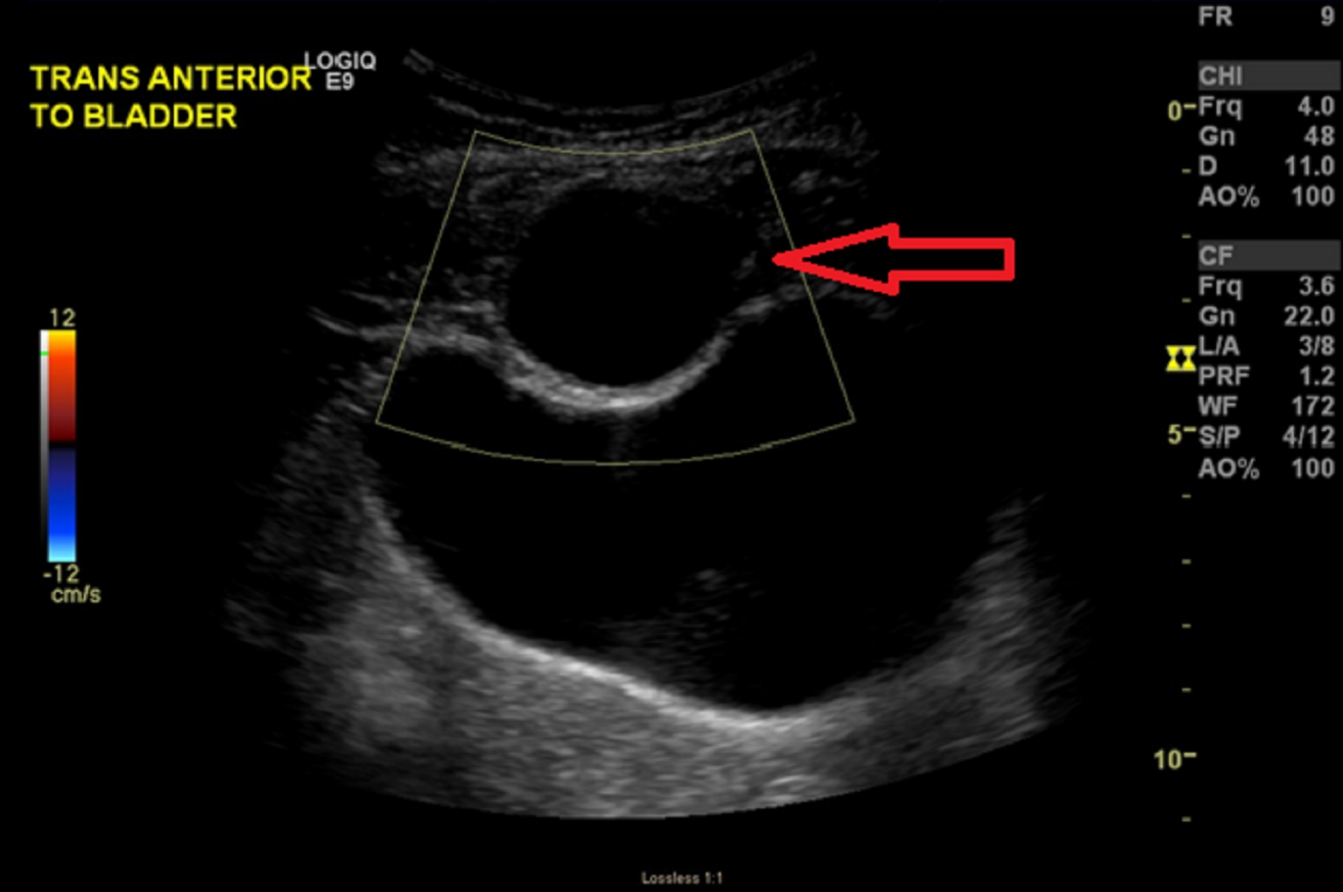
Sonography and Doppler examination of the abdomen revealing a 5 cm x 3 cm heterogeneous hypoechoic mass (arrow)

**Figure 2:**
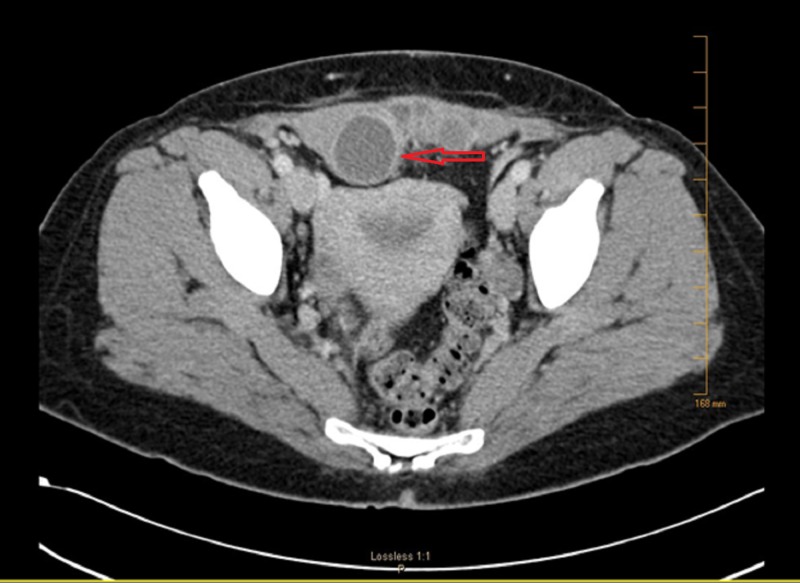
Axial computed tomography scan revealing a 6.2 x 6.8 x 3.6 cm complex cystic lesion involving the rectus muscle (arrow)

Her physical examination revealed a palpable tender subcutaneous mass located under the incision scar. Sonography and Doppler examination of the abdomen revealed a 5 cm x 3 cm heterogeneous hypoechoic mass, with an otherwise normal examination of the uterus and ovaries (Figure 1). Computed tomography (CT) scan revealed a 6.2 x 6.8 x 3.6 cm complex cystic lesion involving the rectus muscle and fascia without traversing the peritoneum (Figure 2).

Fine needle aspiration revealed amorphous debris, hemorrhage, and hemosiderin cystic degeneration, negative for malignant cells. An ultrasound-guided core needle biopsy of the abdominal wall mass was conducted by an interventional radiologist, and pathology revealed benign endometrial glands and stroma compatible with endometriosis with active inflammation and fibrosis. Test results were negative for epithelial dysplasia.

**Figure 3:**
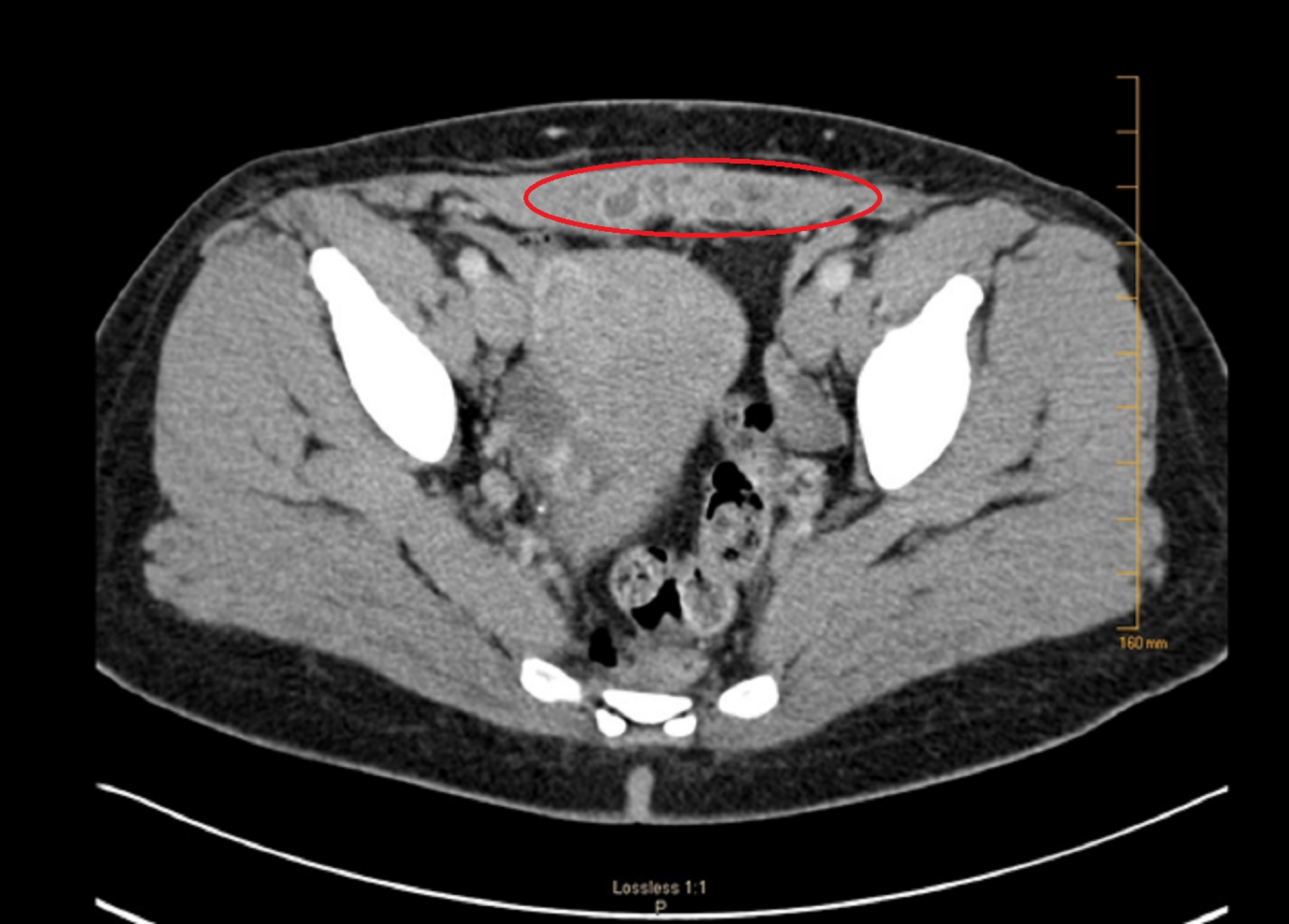
Six-month follow-up axial computed tomography scan revealing marked shrinkage of the abdominal mass (oval ring) while on hormonal therapy

She was managed with hormonal therapy (Lupron®). Six months into treatment with hormonal therapy, her abdominal pain, and vaginal bleeding resolved, and she had no awareness or feeling of an abdominal mass. A CT scan revealed marked shrinkage of the abdominal mass (Figure 3).

**Figure 4:**
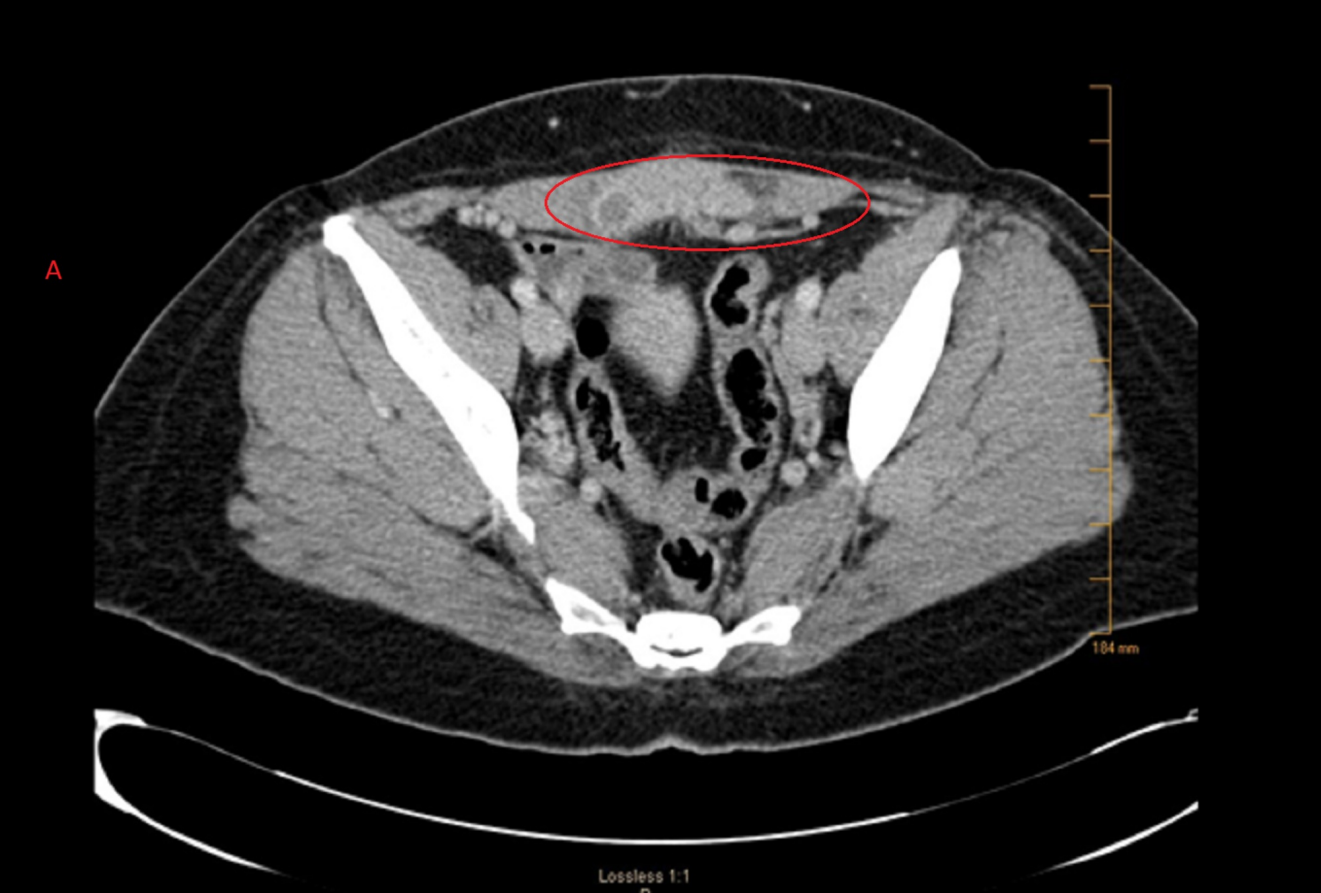
At 12-month follow-up, patient was two months off hormonal therapy, and axial computed tomography scan revealed abdominal mass remained shrunken (oval ring)

At the 12-month follow-up, the patient was discontinued on hormonal therapy for approximately two months, her menses were regular, and she had no abdominal pain. A CT scan revealed further shrinkage of the abdominal mass (Figure 4).

At that time, we discussed options such as observation, radiation therapy, or surgical resection. Surgical resection would have been an extensive procedure given the location and a full-thickness involvement of the medial aspect of the bilateral rectus muscles. The patient elected to continue with observation and follow-up every six months with a CT scan.

## Discussion

Due to its asymptomatic nature in some women, determining the prevalence of ectopic endometriosis can be a daunting task. However, surgery is the definitive diagnostic means [2]. Endometriosis affects approximately 6% to 10% of women of reproductive age. Patients usually present with dysmenorrhea, dyspareunia, chronic pelvic pain, and infertility [3]. The incidence of affected women who experience pain, infertility, or both can be as high as 35% to 50% [4].

In decreasing order of frequency, the most common anatomic sites for this lesion are ovaries, posterior and anterior cul-de-sac, posterior broad ligaments, uterosacral ligaments, uterus, fallopian tubes, sigmoid colon, and appendix and round ligaments [5].

Associated risk factors for ectopic endometriosis include nulliparity [6] and prolonged exposure to endogenous estrogen, such as early menarche or late menopause [7]. Heavy menstrual bleeding, obstruction of menstrual outflow, and high consumption of transunsaturated fats are also risk factors [8]. Risk factors for developing surgical scar endometriosis can include women who have undergone prior abdominal or pelvic operation, hysterotomy in the second or third trimester, alcohol consumption, increased menstrual flow, and obesity [1,8,9].

Sonography and Doppler examinations can reveal heterogeneous hypoechoic mass with echogenic spots. Diagnosis can be confirmed with excision and analysis of tissue samples from the area, and examination will reveal fibrosis of the tissue that involves the fascia of the area as well [9].

Surgery is the definitive treatment for endometriosis and must be performed with clear margins and followed up with reconstruction of the damaged tissue. Hormone suppression has also been suggested since hormone induction due to surgical procedure is a suspected cause of the condition [9]. Preventive measures include a thorough isolation of the incision site and cleaning of the pelvic cavity before closure of the abdominal wall postsurgery [9].

## Conclusions

Surgical scar endometriosis is a rare condition that is difficult to diagnose. Surgery is the definitive diagnosis and management. However, in carefully selected patients, hormonal suppression has been used to successfully manage this condition, as evidenced in our case presentation.
